# Determination of the Genetic Component of Fur-Chewing in Chinchillas (*Chinchilla lanigera*) and Its Economic Impact

**DOI:** 10.3390/ani8090144

**Published:** 2018-08-21

**Authors:** Catalina González, José Manuel Yáñez, Tamara Tadich

**Affiliations:** 1 Departamento de Medicina Preventiva Animal, Facultad de Ciencias Veterinarias y Pecuarias, Universidad de Chile, Santa Rosa 11735, La Pintana, Santiago 8820000, Chile; cata2403@gmail.com (C.G.); jmayanez@uchile.cl (J.M.Y.); 2 Departamento de Fomento de la Producción Animal, Facultad de Ciencias Veterinarias y Pecuarias, Universidad de Chile, Santa Rosa 11735, La Pintana, Santiago 8820000, Chile

**Keywords:** chinchilla, welfare, fur-chewing, abnormal behavior, fur price, heritability

## Abstract

**Simple Summary:**

Many chinchillas kept in captivity develop fur-chewing. This behavior does not only affect fur price, but most importantly it can be the result of an animal welfare problem. The causes of this behavior are not well understood and a genetic component could exist. This is why the aim of this study was to determine the genetic component and the effect of this behavior on fur price. The data from a commercial fur-farm was used, it included information on 10,196 chinchillas recorded between 1990 and 2011. The heritability of the behavior and its effect on fur price were determined. The results show a significant genetic variation in fur-chewing with an estimated heritability of 0.16. At the same time, the behavior had an important negative effect on fur price. The selection and management practices used in fur-farming should be improved in order to decrease the incidence of this behavior.

**Abstract:**

Fur-chewing is a common behavioral disorder developed by chinchillas kept in confinement that can indicate a past or present welfare problem. It also has a negative productive impact associated. The aim of this study was to determine the genetic component of fur-chewing, and the effect of this undesired behavior on fur price in a commercial fur-farming system of chinchillas (*Chinchilla lanigera*). The data for the analysis was derived from a commercial population of 10,196 chinchillas, recorded between the years 1990 and 2011. For determining differences in fur price according to presence of fur-chewing behavior, analysis of variance (ANOVA) was used, considering 3007 animals. For estimation of variance components of fur-chewing a sire-dam threshold (probit) mixed model was used, using data of 9, 033 individuals, and then heritability on the underlying liability scale was calculated. The analysis revealed a significant negative impact on fur price from fur-chewing chinchillas (*p*-value < 0.05). In addition, the study showed that fur-chewing presents significant genetic variation, with an estimated heritability of 0.16. The presentation of fur-chewing should be taken into account when selecting broodstock in these systems, in order to reduce the number of affected individuals.

## 1. Introduction

Over the past century to date, the multimillionaire fur industry has been developed through the breeding of fur animals, like minks (*Neovison vison*), ferrets (*Mustela putorious*), rabbits (*Oryctolagus cuniculus*), otters (*Myocastor coypus*), foxes (*Alopex lagopus, Vulpes vulpes*), among others. The hystricomorphic rodent Chinchilla (*Chinchilla lanigera*) is a very important part of the fur trade [1]. Chinchillas are endemic of Chile and it is possible to find two species in the wild, *Chinchilla brevicaudata* and *Chinchilla lanigera*, in both cases there is limited knowledge about their biology and history. Additionally, there are limited studies about the physiology of chinchillas kept in captivity [2,3].

Chinchillas bred for fur-farming originated as a hybrid, product of the cross-breeding of both taxa 80 years ago. However, *Chinchilla lanigera* was selected over time to originate the captive chinchillas found nowadays [4]. Although *C. brevicaudata* presented a denser and longer fur [5], *C. lanigera* is characterized for being more docile, with a briefer gestation period and a greater quantity of offspring per birth, therefore being more suitable for fur production systems [6,7,8]. In the fur industry, chinchillas have a great commercial value due to the exceptional qualities of their fur, which reaches in average 2.5 cm of length, is silky, extremely soft, and firmly attached to the skin. In most mammals each hair follicle produces only one hair, but in this species each follicle produces between 24 and 80 hairs [9].

A widely studied behavior in the chinchilla fur industry, due to its important economic impact, is fur-chewing. This repetitive abnormal behavior performed by chinchillas kept in captivity has been studied due to its negative physiological and productive consequences [10]. Stereotypes are defined as a behavioral disorder, where the behavioral patterns are invariant and repetitive, with no obvious aim or function [11]. The abnormal repetitive behaviors can increase when non-human animals are confined in barren environments that lack sensorial or cognitive stimulation, and/or for being constantly exposed to an adverse stimulus. These behaviors can be even more pronounced when the animal cannot perform behaviors that they are highly motivated to do in an open-range environment, and that they would normally do as they are essential for their survival in the wild [12].

It has been described that sometimes these behaviors seem to have an adaptive function for the individual, allowing them to cope with captivity. Therefore, it has been noted that the development of these behaviors is a direct consequence of suboptimal environments, where animals have no control, generating situations of frustration, stress, fear, and lack of stimulation. Stereotypes can have damaging effects on animals’ health, as well as over their reproductive and productive performance [13,14].

Due to the previously mentioned negative effects, direct behavioral indicators of animal welfare, such as the presentation of stereotypes, are taking more relevance. Most undesired behaviors are a consequence of animal welfare deterioration; therefore, they should be prevented as much as possible [15,16]. Fur-chewing is a behavioral disorder, classified within the self-mutilation group, in which chinchilla either continuously or intermittently chew their own fur from the lumbar area down to the tail [17]. The behavior is more frequent in females, but can develop in both males and females usually starting at around 6–8 months of age. Fur-chewing does not only affect the animal’s welfare, but also affects their skin, with the potential to impact large areas of their body. In consequence, affecting the fur quality, it also affects the fur price, since size and quality are the features that influence fur price the most [18,19]. Studies undertaken in Argentina and Chile have reported a prevalence of the behavior of approximately 4% of the captive chinchilla population [17,20].

Most studies on fur-chewing have been directed towards the association with management of some environmental factors, relegating other factors such as biological and individual characteristics. The possibility that fur-chewing is a behavior with a heritable component is usually discussed among breeders [21]. It has been demonstrated through experiments made by Mösslacher in 1986 [22] that up to 50% of the offspring of females presenting the behavior have also turned into animals that perform fur-chewing. It has been postulated that genetic lines with a predisposition to be more affected by the stress generated by captivity may exist, and therefore be predisposed to develop the behavior. However, more studies are required to affirm the presence of a genetic component involved in fur-chewing [17,23].

Research in equines postulates a heritable base for some stereotypes; a heritability value of 0.68 has been recently estimated for crib-biting [24]. This gives reason to speculate that the presence of susceptible genes is required in an individual genome, before the environmental stimuli can cause the apparition of the behavior [25]. Furthermore, a heritability estimate for stereotyped behaviors of 0.25 is given in a study performed in minks [26]. However, to our knowledge, currently there are no studies reporting heritability estimates for fur-chewing in chinchillas. Likewise, there are no studies revealing quantitatively the economic impact that fur-chewing would have over fur price. This information would allow developing genetic selection programs that could favor both the welfare of chinchillas and the profitability of the productive system.

The aim of the present study was to determine the possible influence of fur-chewing over the fur price in chinchillas, and to determine the genetic component of the behavior, in a commercial chinchilla fur-farming system.

## 2. Materials and Methods

### 2.1. Data

The dataset comprised records from 10,196 chinchillas, covering from year 1990 until 2011. To analyze the relation between fur-chewing and fur price, considering sex and slaughter year, data from 3007 animals were used, and to estimate heritability, information of fur-chewing, sex, slaughter weight, sire and dam of 9033 individuals were used. Animals belonged to a commercial breeding facility located in Pirque, Metropolitan Region, Chile.

### 2.2. Statistical Analysis

Differences in fur price depending on the presence or absence of fur-chewing were evaluated using the following analysis of variance (ANOVA) model fitted using Infostat statistical software. This analysis was done with 3007 animals, which had complete information on fur price, sex, fur-chewing and slaughter year.

The dependent variable animal’s fur price (USD) was analyzed using fur-chewing (1 = presence; 0 = absence), sex (male and female) and slaughtering year as independent variables. Fur-chewing and sex were considered as factors and the slaughtering year as a covariate. All the independent variables were considered fixed effects.

### 2.3. Heritability

This analysis was done with 9,033 animals, which had complete information on fur-chewing, sex, slaughter weight, sire and dam of each individual. In order to estimate the variance components involved in the fur-chewing behavior, the data was analyzed using a sire-dam threshold (probit) mixed model. The phenotypic observations of fur-chewing in chinchillas were recorded as a binary character, were (0) are animals that did not perform the behavior, and (1) are those that were observed performing it. The binary threshold (probit) model used was as it follows:

$$\;Pr(Y_{ijkl})=\Phi \left(\mu +S_{i}+FW_{ijkl}+F_{j}+\;M_{k}\right)\;$$

where:

$Y_{ijkl}$ = the observation of presence/absence (1 or 0) for fur-chewing of the individual l;
Φ = cumulative standard normal distribution;
μ = fixed effect of the general mean;
$S_{i}$ = fixed effect of sex *i* (*i* = male or female);
$FW_{ijkl}$ = fixed effect of final weight of the individual l;
$F_{j}$ = random effect of the sire *j* (*j* = 459);
$M_{k}$ = random effect of the dam *k* (*k* = 1320);

The parameters of the threshold univariate model were estimated through restricted maximum likelihood using the statistical software ASREML^®^ 3.0 (NSW Department of Industry and Investment, Australia) [27]. The implicit residual variance on the underlying liability scale for the threshold (probit) model was set to 1 [28].

The values of sire and dam additive variance and residual variance, were used to calculate the heritability of fur-chewing using the following formula:

$$\;h^{2}=\frac{2\;(\sigma_{a}^{2}+\;\sigma_{m}^{2})}{\sigma_{a}^{2}+\;\sigma_{m}^{2}\;+\sigma_{e}^{2}}\;$$

where:

$h^{2}$ = heritability of fur-chewing;
$\sigma_{a}^{2}$ = additive variance of the father for fur-chewing;
$\sigma_{m}^{2}$ = additive variance of the mother for fur-chewing;
$\sigma_{e}^{2}$ = residual variance for fur-chewing (set to 1).

## 3. Results

Regarding sex distribution, 47% of chinchillas in the study were females, and 53% were males. Every year there was a higher percentage of males with regard to females, excluding year 2011, when there was the same percentage of animals of both genders. Chinchillas in the present study had a mean weight of 590.5 grams at sacrifice, and their mean fur price was 40.6 American dollars each (Table 1).

In relation to the presentation of fur-chewing, overall 5% of chinchillas registered the behavior, from these 49.9% where females and 50.1% were males. Variations in the percentages can be observed throughout the years, with a range between 2% for years 1994 and 1995, up to 9% registered in 2010.

A total of 459 sires were registered in the dataset, many of whom remained in the productive system for more than one breeding year. Sires had a mean of 22.13 offspring each, having a range between 1 and 298 offspring per father. For dams, a total of 1320 records were identified, who had a mean of 7.7 (between 1 and 44) offspring per dam.

The results of the analysis of 3007 animals, show that there is a significant difference (*p*-value < 0.001) in the fur price of chinchillas that present fur-chewing, in comparison to those that did not perform that stereotype. The mean price of the fur of fur-chewing individuals, adjusted posterior to the ANOVA analysis was US$21.1, in comparison to a mean of US$38 dollars obtained by the furs of chinchillas that did not perform the behavior. It also shows that there is a statistically significant (*p*-value < 0.05) influence of sex and year of slaughter over fur price (Table 2). The mean price of the fur of females was US$28.6, in comparison to a mean of US$30.5 dollars obtained by male furs.

The estimate of the genetic variance of the sire, the phenotypic variance and the heritability of fur-chewing are significant, however, the dams estimated component was not, since it is lower than twice its standard error. The heritability estimate for fur-chewing behavior was 16% as it is shown in Table 3.

## 4. Discussion

Fur-chewing has detrimental welfare implications, but also negatively impacts the fur production system by significantly decreasing the value of the furs, as was estimated in the present study. It also was found that the behavior has a genetic component, which is of great relevance for possible further artificial selection programs.

The population studied presented sex, weight at sacrifice, and fur price distributions similar to previous studies [19]. For example, the nearly 1:1 sex ratio observed in the present study is similar to the ones reported by García [29]. In the same line, the mean weight at sacrifice of the animals in this study is in agreement with the values shown in previous studies on chinchillas [30,31], although slightly lower to those reported by Sportono et al. [32]. Breeders have been selecting chinchillas mainly on size (which is directly associated to weight) and color, since these are the two characteristics that most influence the final prices of individual furs [19] and could explain the similarity in the variables studied across countries.

The percentages of presentation of fur-chewing in the studied chinchillas, are also in accordance with prior investigations [17,20,21] and had the same frequency of presentation among females and males. Although Ponzio et al. [17] reported a higher incidence in females, it has to be taken into account that their results correspond to a survey, where producers were asked if according to their criteria one sex developed fur-chewing more than the other, while our study reports the animals that were classified as fur-chewers according to fur damage. Regarding the association between fur chewing and fur price, it should be noted that previous studies mention the relevance of this behavior for the fur industry due to its economic repercussions, either because individuals are eliminated from the system or because of a decrease in the quality of the final product [12,17,33]. However, those studies did not quantify the price difference between furs originated from individuals that developed the behavior, in comparison to those that did not. In the present study, a significant price difference was found, with a mean fur price of US$21.1 for those that come from fur-chewing individuals compared to US$38 from those that did not develop this abnormal repetitive behavior. Although the prevalence of fur-chewing could seem rather low (5%), it has to be taken into account that in the fur industry the price is set per individual fur, thus the economic impact can be of importance [4], in this case a reduction of 55.5% of the price per fur. Furthermore, individuals that perform the behavior might be eliminated from the system at an early stage, not reaching the sacrifice age and resulting in complete loss of the product. This should be carefully analyzed in terms of the welfare of these chinchillas. The WelFur Project^®^ aimed to develop on farm welfare assessment protocols for minks and foxes in the fur industry, taking as a base the Welfare Quality Project [15]. Although the project does not include chinchillas, many of the welfare indicators used, including the assessment of the presentation of stereotyped behaviors could be applicable in chinchilla fur industry, as a mean of improving their overall welfare.

Some studies suggest that chinchillas with fur-chewing are particularly sensitive to stress. It has been suggested that the condition has a heritable component [7], and that the inability of some individuals to cope with the stress of captivity could be genetically transmitted. This idea is strengthened by the fact that not all animals that experience suboptimal or stressful environments demonstrate self-mutilating or other abnormal repetitive behaviors [34]. However, there are no previous studies providing heritability estimates for fur-chewing for chinchillas. In this study, we used a threshold (probit) mixed model to estimate the heritability of fur-chewing behavior, in order to account for the binary nature of this trait. Sire and dam variance components were estimated to calculate heritability given that for most of the families’ pedigree was not linked across generations, resulting in an estimated heritability of 0.16. On the other hand, there are some studies ensuring the existence of a heritable component in other abnormal repetitive behaviors, where the fur/feathers are removed by the individuals themselves, which have been related to fur-chewing in terms of phenomenology and etiology, for example, barbering in mice, where it has been noted that genetics play an important role; similarly, it has been demonstrated that trichotillomania has a heritable component in people [33,34]. In addition, it has been noted in minks, stereotyped behavior and fur-chewing have a genetic component in the mink’s predisposition to perform them [35]. Furthermore, the heritability in stereotyped behaviors in minks was estimated, resulting in 0.25 [26], which is higher than the result obtained in the present study (0.16). More recently the heritability for crib-biting in equines was estimated, obtaining higher values (0.68) than those of the present study [24].

The genetic predisposition to fur-chewing has also been found in other species of animals. In a study involving mice, a mutation in gen Hoxb8 was considered as a genetic factor behind fur-chewing. When kept separated, the mice with mutation of this gene would pull out or eat excessively their fur [21]. It has been noted in humans, that the generalized anxiety disorder also possesses a genetic factor, where the first degree relatives of affected people present a risk of 20% (much higher than the 5% of the general population) of presenting the disorder [36]. On the other hand, it should be noted that the existence of a genetic component in complex traits does not mean that the environment is not important. In the complex characters, the environmental influence is normally as important as genetics. The genetic influence on behavior is just a factor, not something preprogrammed or deterministic [36,37]. Even high heritability’s only indicate that the current environmental influences do not seem to greatly affect that trait, although other experiences could [38].

Under this perspective, the environmental factors become particularly important. It is theorized that the genetic predisposition to develop abnormal repetitive behaviors is expressed when the animals are exposed to certain environmental experiences, which can be associated to inappropriate environments, early or abrupt weaning, or other forms of stress during development [34,39,40]. In addition, it has been demonstrated that development in a barren environment leads to permanent changes in the central nervous system (CNS). Animals forced to live in a sterile environment during early weeks, months or years of their development, have demonstrated to have less neurons in their brain, lower dendritic bifurcation, and spinal density, and reduced synaptic connectivity, besides from a higher incidence of abnormal repetitive behaviors. This discovery leads to the complex and multifactorial base of these problems, reason why they can be so hard to prevent or treat [34]. In the present study, records from Chinchillas since 1990 were used to estimate heritability, environmental factors that have been described as risk factor for the presentation of this behavioral disorder were not taken into account since they were not available. A future prospective study should consider estimating the heritability of the behavior under different environmental conditions such as housing (cage position within the system), lighting, and noise, in order to determine if the expression of this behavior varies.

Long-term exposition to stress can lead to the development of repetitive behaviors induced by frustration, repetitive attempts to cope and/or CNS dysfunction, known as obsessive compulsive disorder [41]. These animals will deviate from the regular behavior patterns that are typically found at a given age and sex in a more natural environment [21]. In minks, the development of stereotypes is influenced by the environment and the routine managements, such as feeding strategies (hunger) [42]. In a study made by Malmkvist et al. [43], a reduction of stereotyped behavior was noticed when thick food was provided, increasing the consummatory element of their daily foraging. In the same study, fur-chewing was reduced in female minks with access either to biting ropes or to thick food. Besides the unsatisfied foraging elements leading to abnormal behavior, it has been suggested that fur-chewing could be related to low stimulation, leading to over-expression of other behaviors such as grooming. According to this, an increase in the daily stimuli could be the reason why biting ropes as well as the thick food reduced fur-chewing [43]. It has also been observed stress induced fur-chewing in degus (*Octodon degus*), resulting from loneliness of the animals in their individual housing [21]. Ponzio et al. [12,17] found that factors such as noise, lack of companion or incompatible companion, appearance of an unknown person to the farm, and boredom, predisposed chinchillas to develop fur-chewing behavior. Contrary, the use of sawdust as bedding material maintains chinchillas engaged in positive behaviors and reduced the appearance of the behavior [44].

Thus, environmental enrichment could be a positive management measure that provides animals with opportunities to perform specie-specific behaviors, and gives them some control over their environment whenever possible. It has been demonstrated that being able to control certain aspects of their surroundings (for instance, nest construction for thermoregulation) diminishes stress for captivity conditions for many species. In addition, all efforts must be made to ameliorate aspects of the environment that the animal finds stressing [34].

Finally, with the results of the present study, the possibility to carry out genetic selection beyond production traits, given that the heritability is low and significant, could decrease the percentage of fur-chewing presentation within the system. With this, besides increasing the establishment’s profitability, the animals’ welfare within the fur production system could also be increased, by stopping the reproduction of individuals with a predisposition to stress generated by captivity [45,46]. Breeding for behavior has already been proposed as an opportunity for improving animal production and welfare, which requires the development and validation of proxy measures of key behavioral traits [47].

## 5. Conclusions

In conclusion, fur-chewing behavior has an effect over fur price, and the reported genetic parameter in the present study indicated that a genetic improvement of this trait can be accomplished (low and significant heritability for fur-chewing). These results raise the possibility of selection based on the presentation of the stereotyped behavior, which coupled with an environmental enrichment program, could diminish fur-chewing presentation therefore increasing not just average fur price of chinchillas, but also the animals’ welfare.

## Figures and Tables

**Table 1:** Number of observations (*N*), mean, standard deviation (S.D.), variation coefficient (VC), minimum value (Min) and maximum value (Max) for each trait, considering fur-chewing (0 = not fur-chewer; 1 = fur-chewer) and sex (F = female; M = male) as categories.

	Fur-Chewing	Sex	*N*	Mean	S.D.	VC	Min	Max
Slaughtering weight *	0	F	1141	593.5	85.6	14.4	280	891
		M	993	585.6	80.9	13.8	313	870
	1	F	63	612.0	77.5	12.7	372	808
		M	67	592.3	81.2	13.7	406	817
Total/Mean			2264	590.5	83.2	14.1	280	891
Fur price **	0	F	1226	40.1	15.4	38.5	0	100
		M	1644	42.3	14.6	34.6	0	100
	1	F	57	23.2	11.9	51.2	0	54
		M	80	25.5	13.2	51.6	0	70
Total/Mean			3007	40.6	14.9	36.9	0	100

* Slaughtering weight is registered in grams; ** fur price is registered in US dollars.

**Table 2:** Analysis of variance (ANOVA) of fur-chewing, animal sex and year of slaughter, over fur price in chinchillas.

SV ^1^	SS ^2^	DF ^3^	MS ^4^	F ^5^	*p*-Value
Model.	47,825.9	3	15,941.9	72.9	<0.0001
Fur-chewing	36,973.2	1	36,973.2	169.0	<0.0001
Sex	3351.2	1	3351.2	15.3	0.0001
Year	7501.5	1	7501.5	34.3	<0.0001
Error	656,702.5	3003	218.7		
Total	704,528.4	3006			

^1^ SV = Source of variation; ^2^ SS = Sum of squares; ^3^ DF = Degrees of freedom; ^4^ MS = Mean square; ^5^ F = *F*-statistic column.

**Table 3:** Genetic variance estimates of the father ($\sigma_{a}^{2}$), genetic variance of the mother ($\sigma_{m}^{2}$), phenotypic variance ($\sigma_{p}^{2}$) and heritability ($h^{2}$) with their standard errors (S.E.) for fur-chewing in chinchillas.

Variable	$\sigma_{a}^{2}\;\pm \;S.E.$	$\sigma_{m}^{2}\;\pm \;S.E.$	$\sigma_{p}^{2}\;\pm \;S.E.$	$h^{2}\;\pm \;S.E.$
Fur-chewing	0.044 ± 0.017	0.042 ± 0.022	1.087 ± 0.024	0.160 ± 0.042
